# Distance-limited walk tests post-stroke: A systematic review of measurement properties^1^

**DOI:** 10.3233/NRE-210026

**Published:** 2021-06-16

**Authors:** Darren Kai-Young Cheng, Matthieu Dagenais, Kyla Alsbury-Nealy, Jean Michelle Legasto, Stephanie Scodras, Gayatri Aravind, Pam Takhar, Erica Nekolaichuk, Nancy Margaret Salbach

**Affiliations:** a Department of Physical Therapy, University of Toronto, Canada; b Rehabilitation Sciences Institute, University of Toronto, Canada; c Michener Institute of Education, University Health Network, Canada; d KITE-Toronto Rehabilitation Institute, University Health Network, Canada; e Gerstein Science Information Centre, University of Toronto, Toronto, ON, Canada

**Keywords:** Gait, rehabilitation, stroke, assessment, measurement properties

## Abstract

**BACKGROUND:**

Improving walking capacity is a key objective of post-stroke rehabilitation. Evidence describing the quality and protocols of standardized tools for assessing walking capacity can facilitate their implementation.

**OBJECTIVE:**

To synthesize existing literature describing test protocols and measurement properties of distance-limited walk tests in people post-stroke.

**METHODS:**

Electronic database searches were completed in 2017. Records were screened and appraised for quality.

**RESULTS:**

Data were extracted from 43 eligible articles. Among the 12 walk tests identified, the 10-metre walk test (10mWT) at a comfortable pace was most commonly evaluated. Sixty-three unique protocols at comfortable and fast paces were identified. Walking pace and walkway surface, but not walkway length, influenced walking speed. Intraclass correlation coefficients for test-retest reliability ranged from 0.80–0.99 across walk tests. Measurement error values ranged from 0.04–0.40 and 0.06 to 0.20 for the 10mWT at comfortable and fast and paces, respectively. Across walk tests, performance was most frequently correlated with measures of strength, balance, and physical activity (*r* = 0.26-0.8, *p* < 0.05).

**CONCLUSIONS:**

The 10mWT has the most evidence of reliability and validity. Findings indicate that studies that include people with severe walking deficits, in acute and subacute phases of recovery, with improved quality of reporting, are needed.

## 1 Introduction

Use of standardized assessment tools is considered a best practice in stroke rehabilitation to evaluate the magnitude of gait deficit, monitor response to therapeutic intervention, educate, and set patient-centered goals (Moore et al., 2009; Otterman et al., 2017; Potter et al., 2011; Teasell et al., 2020). Distance-limited walk tests, such as the 10-metre walk test (Wade, 1992) (10mWT), have been recommended for assessing gait speed after stroke (Kwakkel et al., 2017; Otterman et al., 2017; Sullivan et al., 2013; Teasell et al., 2020). Gait speed is an important outcome of stroke rehabilitation as it is essential for community ambulation (Potter et al., 2011; Salbach et al., 2014), associated with motor function, balance (Ahmed et al., 2003; Kwong et al., 2016), walking function (Ahmed et al., 2003; Fulk et al., 2008), and health-related quality of life (Khanittanuphong & Tipchatyotin, 2017), and a predictor of survival (Studenski et al., 2011). Clinical use of measures of gait speed is inconsistent and variable across settings (Agyenkwa et al., 2020; Braun et al., 2018; Salbach et al., 2011; Van Peppen et al., 2008). Knowledge translation research, guided by models, theories and frameworks, is needed to overcome barriers to gait speed measurement in clinical practice.

The knowledge-to-action (KTA) framework (Graham et al., 2006) is a knowledge translation framework used to guide the process of translating research into practice. Specifically, the knowledge creation funnel in the KTA framework is used to describe the filtering process required to develop knowledge products or tools for end-users. At the base of the funnel, first-generation knowledge refers to the various individual sources of information on a topic, such as research articles and reports, that are of variable quality and time-consuming to acquire. Second-generation knowledge, or knowledge synthesis, is described as an essential precursor to the development of third-generation, user-friendly knowledge tools such as evidence-based algorithms, guides, and guidelines. PTs report that evidence supporting the measurement properties of standardized tools positively influences their decision to adopt them in clinical practice (Jette et al., 2009; McGlynn & Cott, 2007; Pattison et al., 2015). Therefore, the synthesis and critical appraisal of the measurement literature on distance-limited walk tests is necessary to inform the development of knowledge translation strategies designed to facilitate their use among PTs. Such a synthesis for time-limited walk tests has been reported (Salbach et al., 2017). The objective of this study was to synthesize research evidence of the reliability, measurement error, construct validity, and sensitivity to change for distance-limited walk tests in people with stroke. A secondary objective was to determine the influence of walk test protocol elements on test performance.

## 2 Methods

### 2.1 Overview

A systematic review was conducted in two phases guided by a review protocol developed by the research team. The PRISMA checklist (Liberati et al., 2009) was used to guide reporting. Title and abstract and full text screening forms, the critical appraisal form and data extraction form and guide were piloted and refined prior to use by reviewers. All reviewers involved with study selection and appraisal completed orientation and training with the study coordinator.

### 2.2 Search methods

An initial search was conducted in July 2013 using methods previously described (Salbach et al., 2017) and updated in 2017 due to advancements in literature search methodology (Garner et al., 2016). In collaboration with an academic health sciences librarian, we designed a new Medline search strategy that was peer-reviewed by a second librarian (McGowan et al., 2016), before being translated for use with other databases. The updated search included Ovid MEDLINE: Epub Ahead of Print, In-Process & Other Non-Indexed Citations, Ovid MEDLINE^®^ Daily and Ovid MEDLINE^®^, OVID Embase, EBSCO CINAHL, EBSCO SportDiscus, and *The Cochrane Library* from inception to August 16th, 2017. The new search strategy captured all articles that were included in the original unpublished review. See Supplemental Digital Content 1A for search strategies. A manual search of reference lists and authors’ personal libraries was also conducted.

Records identified in the updated search were imported into EndNote™ software (version X7.7) and duplicate citations were removed using the Bramer method (Bramer et al., 2016). All unique records from the updated search were compared to records found in the original unpublished search, and duplicates, previously screened for eligibility, were removed. The final set of records was uploaded to Covidence™ (https://www.covidence.org) for screening.

### 2.3 Selection criteria

Studies were considered eligible if: (1) participants included adults (18 + years) post-stroke; (2) the study reported on reliability, measurement error, construct validity, and sensitivity to change, or the effect of a walk test protocol element (e.g., walkway length, practice trials, etc.) on performance of distance-limited walk tests (for construct validity, studies reporting associations between walk test performance and other variables, regardless of whether this was framed as validity testing, were included); (3) the study reported the timed, acceleration, and deceleration distance to enable test replication; (4) walk tests were performed separately and were not embedded within another test; and (5) the report was written in English, French or Spanish. Studies were excluded if: (1) the percentage of participants with stroke was below 80%; (2) the walk test was completed on a treadmill; (3) instrumented timing methods (e.g., GaitRite mat, footswitches) were used; or (4) the study was a conference proceeding, dissertation, case report/series or limited to abstract form.

To ensure the feasibility of the review, inclusion of studies examining construct validity was limited to those reporting unadjusted correlations and associated *p*-values or confidence intervals between walk test performance and measures of motor function, aerobic capacity, balance, balance self-efficacy, strength (including force, torque and power), walking, stairs, sit-to-stand, mobility, physical activity, participation, health-related quality of life, or discharge destination as these constructs are considered important rehabilitation outcomes (Lang et al., 2011; Otterman et al., 2017). Among studies examining predictive validity, only those reporting the ability of a distance-limited walk test to predict VO_2peak or max_, physical activity, discharge destination, or health-related quality of life were included. Among studies reporting reliability, only those reporting an intraclass correlation coefficient (ICC) were included. Among studies reporting measurement error, only those reporting minimal detectable change (MDC) and/or standard error of measurement (SEM) were included.

### 2.4 Study selection

Three reviewers screened titles and abstracts independently and in duplicate, and classified studies as potentially relevant or not relevant to the review. Full-texts of potentially relevant records were uploaded to Covidence™ and screened by one of six reviewers to determine eligibility. A second reviewer was consulted to resolve uncertainty regarding the eligibility of a study.

### 2.5 Data extraction

A single reviewer independently extracted data on general study information, study characteristics, participant characteristics, walk test protocol and results from included studies. To ensure data accuracy and completeness, another reviewer randomly selected and verified data from 30% of included articles. Discrepancies were resolved through discussion. Data on participant characteristics (i.e., age, time since stroke onset, sex, type of stroke, side of stroke, walking speed, use of walking aids/orthoses), walk test characteristics (i.e., name, walkway distances, pace, location, timing method, trials, rest interval, scoring, evaluator position/qualifications/training, instructions), and measurement properties, were collected.

### 2.6 Method of quality assessment

The methodological quality of included studies was assessed using the COnsensus-based Standards for the selection of health Measurements INstruments (COSMIN) Risk of Bias Checklist (Mokkink et al., 2018). The tool classifies each measurement property as very good, adequate, doubtful, or inadequate based on the lowest score reported on the corresponding checklist. The research team adapted the checklists and developed a checklist for assessing sensitivity to change based on the format of the COSMIN checklists (see Supplemental Digital Content 1B). Additionally, operational definitions were developed to optimize scoring consistency. For example, for reliability and measurement error, we defined a retest time interval over which patient stability would be assumed for three recovery phases post-stroke as:≤1 day (acute),≤5 days (subacute) and≤3 weeks (chronic) based on results from longitudinal studies of walking (Jørgensen et al., 1995; Richards & Olney, 1996) and research team consensus (Salbach et al., 2017). A single author assessed the methodological quality of included studies, and a second author, not involved in the quality appraisal, was consulted to resolve uncertainty. COSMIN checklists were applied to studies reporting specific measurement properties, not for properties (i.e., MDC) computed using abstracted data.

### 2.7 Data synthesis and analysis

ICC values and associated 95% confidence intervals (CIs) were extracted when reported. The 95% CI is interpreted as the interval that will capture the true ICC value of the population 95% of the time when repeated random samples are drawn from the population (Shrout & Fleiss, 1979). ICC values used to estimate reliability were interpreted as excellent (ICC≥0.75), acceptable (ICC > 0.40 to < 0.75) or poor (ICC≤0.40) (Andresen, 2000). MDC at the 90% confidence level (MDC_90_) was computed for studies reporting test-retest reliability estimates and standard deviation of baseline score using the following equations: 1 SEM = [SD x sqrt(1 - ICC)] (Beaton et al., 2001) and MDC_90_ = [1.645 x SEM x sqrt(2)] (Beaton et al., 2001). Constructs measured to evaluate validity were classified using the International Classification of Functioning, Disability and Health (World Health Organization, 2001) (ICF). We interpreted correlation coefficients as strong (≥0.70), moderate (0.50 to 0.69), weak (0.30 to 0.49) or negligible (< 0.30) (Landis & Koch, 1977). Effect size and standardized response mean values used to estimate sensitivity to change were interpreted as small (0.2), moderate (0.5), and large (≥0.8) (Cohen, 1977). For those studies evaluating torque at multiple points, only peak torque measured using isokinetic dynamometers was reported. Results for reliability, measurement error, validity, and sensitivity to change were presented by time post-stroke classified as acute (< 1 month), subacute (1–6 months), or chronic (> 6 months) (Hatem et al., 2016) using range/interquartile range (or mean/median values if range was not presented). To facilitate comparison between studies, frequency data were converted to percentages, results were converted to a common metric unit, and values were rounded to a consistent decimal place.

## 3 Results

### 3.1 Study selection

**Fig. 1:**
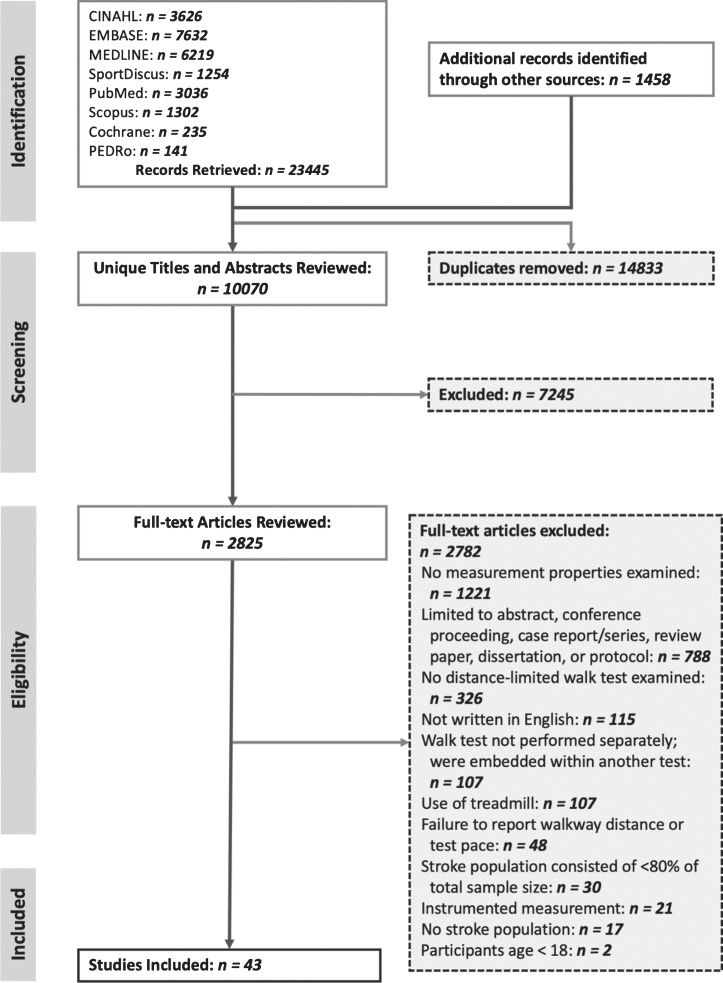
PRISMA flow diagram.

Figure 1 shows the results of the literature search and screening. Of the 24,903 records identified from the 2013 and 2017 searches combined, 10,069 unique records were identified for screening, and 43 articles (Ahmed et al., 2003; Alzahrani et al., 2009; Richard W. Bohannon, 1991; R. W. Bohannon, 1991; Bohannon, 1992; Bohannon & Puharic, 1992; Bohannon & Walsh, 1992; Cheng et al., 2020; Di Cesare et al., 2016; English et al., 2006; English et al., 2007; Ezeugwu & Manns, 2017; Faria et al., 2012; Flansbjer et al., 2006; Flansbjer et al., 2005; Frost et al., 2015; Fulk & Echternach, 2008; Fulk et al., 2008; Fulk et al., 2010; Hiengkaew et al., 2012; Høyer et al., 2014; Huo et al., 2009; Isho & Usuda, 2016; Khanittanuphong & Tipchatyotin, 2017; Kobayashi et al., 2015; Kwong et al., 2016; Lam et al., 2010; Lee et al., 2015; Liu-Ambrose et al., 2007; Mitsutake et al., 2017; Morone et al., 2014; Mudge & Stott, 2009; Nasciutti-Prudente et al., 2009; Ng et al., 2012; Ovando et al., 2011; Peters et al., 2014; Salbach et al., 2013; Salbach et al., 2001; Salbach et al., 2006; Severinsen et al., 2011; Stephens & Goldie, 1999; Taylor et al., 2006; Wang et al., 2014) were included in the review.

### 3.2 Study characteristics

All included articles were written in English. Twelve distance-limited walk tests (identified by walkway distance and pace) were found, including the 3-, 5-, 6-, 7-, 8-, 10-, and 12-metre walk test at a comfortable pace (3-, 5-, 6-, 7-, 8-, 10-, and 12 mCWT, respectively), and the 5-, 6-, 7-, 8-, and 10-metre walk test at a fast pace (5-, 6-, 7-, 8-, and 10 mFWT, respectively). The 10 mCWT was most commonly evaluated (23 articles, 53%). Table 1 presents the number of evaluations of each measurement property, and the effects of walk path length and walking surface on test performance by walk test.

**Table 1:** Frequency of Evaluations of Measurement Properties by Walk Test

Measurement Property or Protocol Element Examined	Number of Evaluations													Number of Articles^1^
	3 mCWT	5 mCWT	5 mFWT	6 mCWT	6 mFWT	7 mCWT	7 mFWT	8 mCWT	8 mFWT	10 mCWT	10 mFWT	12 mCWT	**Total**
Concurrent construct validity	–	5	1	1	–	3	3	2	–	18	5	1	39	32
Reliability	1	2	–	2	1	–	–	–	–	5	4	–	15	11
Measurement error	1	1	–	2	1	–	–	–	–	4	4	–	13	9
Sensitivity to change	–	3	1	–	–	–	–	–	–	1	1	–	6	3
Predictive validity	–	–	–	–	–	–	–	–	–	2	1	–	3	2
Effect of walkway length	–	1	1	–	–	–	–	1	1	1	1	–	6	1
Effect of walkway surface	–	–	–	1	1	–	–	–	–	–	–	–	2	1
Number of Articles^1^	1	10	3	2	1	3	3	3	1	23	12	1

**Abbreviations:** 10 mCWT, 10-metre comfortable walk test; 10 mFWT, 10-metre fast walk test; 12 mCWT, 12-metre comfortable walk test; 3 mCWT, 3-metre comfortable walk test; 5 mCWT, 5-metre comfortable walk test; 5 mFWT, 5-metre fast walk test; 6 mCWT, 6-metre comfortable walk test; 6 mFWT, 6-metre fast walk test; 7 mCWT, 7-metre comfortable walk test; 7 mFWT, 7-metre fast walk test; 8 mCWT, 8-metre comfortable walk test; 8 mFWT, 8-metre fast walk test. ^1^Select articles reported on more than one measurement property and/or more than one test.

### 3.3 Appraisal of study methodology

**Fig. 2:**
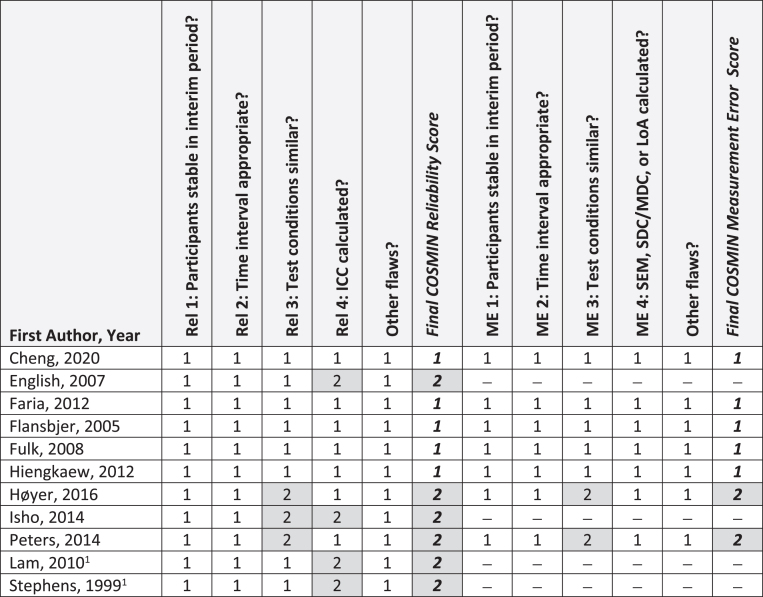
COSMIN Reliability (*N* = 11 articles) and Measurement Error (*N* = 7 articles). **Abbreviations:** ICC, Intraclass correlation coefficient; ME, measurement error; Rel, reliability. **COSMIN Scoring:** 1, very good; 2, adequate; 3, doubtful; 4, inadequate. ^1^Measurement error values were not reported in the article, but computed by study authors using published data; thus, COSMIN checklist was not completed.

**Fig. 3:**
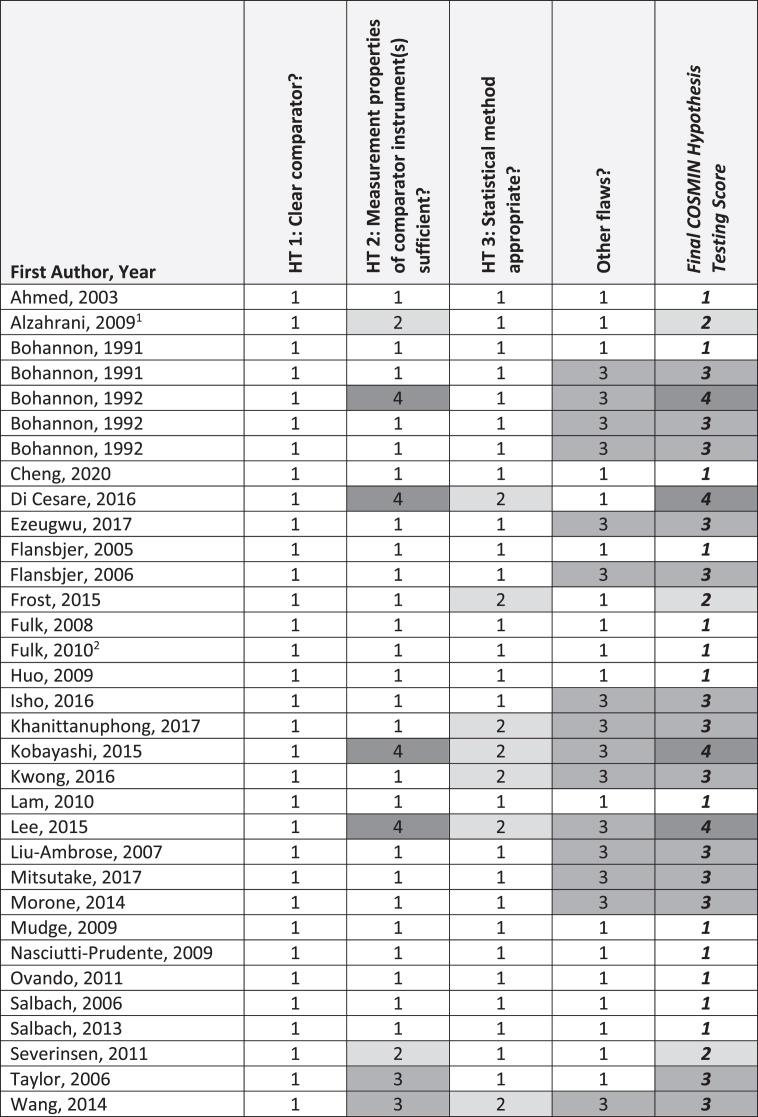
COSMIN Construct Validity (N = 33 articles). **Abbreviations:** HT, hypothesis testing. **COSMIN Scoring:** 1, very good; 2, adequate; 3, doubtful; 4, inadequate. ^1^Evaluated predictive validity only. ^2^Evaluated predictive validity and concurrent construct validity.

**Fig. 4:**
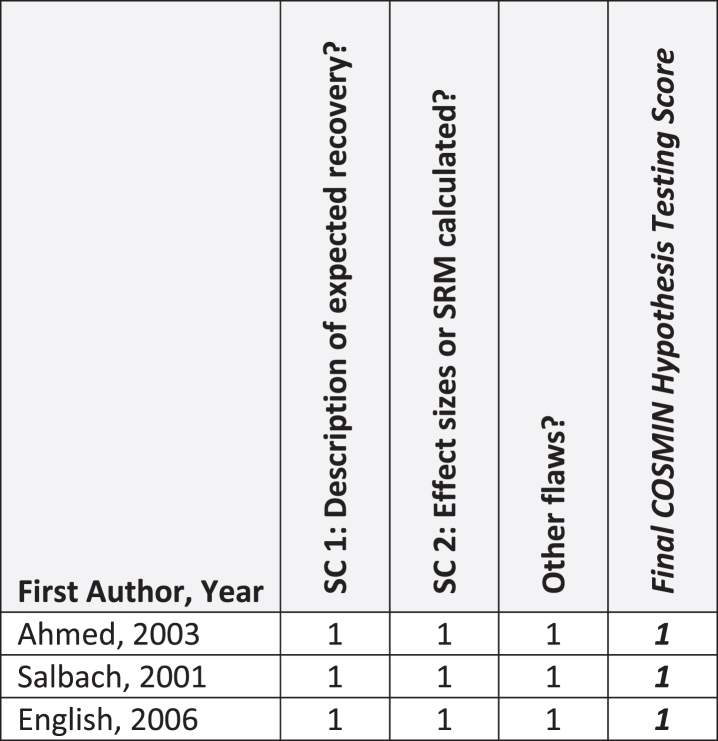
COSMIN Sensitivity to Change (N = 3 articles). **Abbreviations:** SC, Sensitivity to Change; SRM, Standardized response mean. **COSMIN Scoring:** 1, very good; 2, adequate; 3, doubtful; 4, inadequate.

Figures 2, 3, and 4 summarize critical appraisal results for articles assessing reliability and measurement error, construct validity, and sensitivity to change, respectively. All 11 articles evaluating reliability were rated as *very good* or *adequate*. The most prevalent issue was sub-optimal reporting of statistical methods (*n* = 4; 36%). Of the seven articles reporting on measurement error, all were rated as *very good* or *adequate.* The most prevalent issue was sub-optimal reporting of similar testing conditions (*n* = 2, 29%). Of the 33 articles reporting on construct validity, the number rated as very good, adequate, doubtful, and inadequate was 13 (39%), 3 (9%), 13 (39%), and 4 (12%), respectively. The most prevalent issue was other methodological flaws (*n* = 15; 45%), including insufficient descriptions of walk test evaluator position, qualifications, or training received. All 3 articles that evaluated sensitivity to change were rated as *very good*.

### 3.4 Participant and walk test characteristics

The number of articles describing people with acute, subacute and chronic stroke was 2 (5%), 6 (14%) and 21 (49%), respectively. Fourteen studies (33%) included participants in different phases. There were 43 evaluations of walk tests at a comfortable pace and 20 at a fast pace. The position of the evaluator was reported for 15 walk test protocols (24%) as beside (9 protocols (Alzahrani et al., 2009; Cheng et al., 2020; Fulk et al., 2008; Mudge & Stott, 2009; Salbach et al., 2001; Stephens & Goldie, 1999)) behind (5 protocols (English et al., 2006; English et al., 2007; Høyer et al., 2014; Ng et al., 2012)), and beside or behind as needed (1 protocol (Salbach et al., 2013)). Use of assistive devices was reported in 28 articles (65%). Eight protocols (13%) allowed physical assistance to walk. In 25 of 30 articles (83%) that named the walk test administered, the convention was to name the walk test according to the timed distance (e.g., for the 10-metre walk test, time taken to walk 10m is documented). Supplemental Digital Content 2 includes summaries of participant characteristics across articles and details of the 63 unique protocols for 12 walk tests.

### 3.5 Influence of walk test protocol elements on test performance

#### 3.5.1 Effect of walkway length and walking pace

One study (Ng et al., 2012) of 25 participants with chronic stroke did not find significant differences in performance on the 5 m, 8 m, or 10 m walk tests at comfortable or fast pace, indicating these walkway lengths yield similar speeds. Performance at a comfortable pace (mean 0.76–0.79 metres per second (m/s)) was significantly slower than performance at a fast pace (mean 0.97–1.00 m/s) for each walkway length.

#### 3.5.1 Effect of walkway surface

In one study, the effects of walkway surface on 6 mCWT and 6 mFWT performance among 24 people with subacute stroke was examined (Stephens & Goldie, 1999). Participants walked significantly faster on parquetry (hardwood) than on carpet with a mean difference of 0.05 m/s and 0.03 m/s for the 6 mCWT and 6 mFWT, respectively.

### 3.6 Reliability and measurement error

Reliability and measurement error were reported in 11 and 7 articles, respectively, and reported study data were used to calculate measurement error for another 2 articles (Table 2). Intra-rater, inter-rater, and test-retest reliability was reported in 3, 2, and 8 articles, respectively. In 8 studies evaluating 3 mCWT, 6 mCWT, 6 mFWT, 10 mCWT and 10 mFWT protocols that did not allow physical assistance to walk (English et al., 2007; Faria et al., 2012; Flansbjer et al., 2005; Hiengkaew et al., 2012; Isho & Usuda, 2016; Lam et al., 2010; Peters et al., 2014; Stephens & Goldie, 1999), ICC point estimates and lower 95% confidence interval (CI) limits exceeded 0.75. In 3 studies evaluating 5 mCWT, 10 mCWT, and 10 mFWT protocols that allowed the evaluator to provide physical assistance (Cheng et al., 2020; Fulk & Echternach, 2008; Høyer et al., 2014), ICC point estimates exceeded 0.75; in two studies reporting CIs (Cheng et al., 2020; Fulk & Echternach, 2008), lower 95% CI limits were in the acceptable range. Table 2 presents the SEM, smallest real difference, and computed or reported MDC values for the 3 mCWT (*n* = 1), 5 mCWT (*n* = 1), 6 mCWT (*n* = 2), 6 mFWT (*n* = 1), 10 mCWT (*n* = 4), 10 mFWT (*n* = 4).

**Table 2:** Reliability and Measurement Error (*n* = 11 articles; 28 protocols)

First Author, Year	Walk Test	Level of Gait Speed Deficit (m/s)	Walk Test Protocol			Re-test Time Interval	N	Test-Retest Reliability ICC (95% CI) Unless Otherwise Stated	Measurement Error	COSMIN Quality Score
			TD, AD, DD	Practice Trials, Test Trials, Position of Rater, Timing Tool	Pace, Walking Aids Allowed, Assistance Allowed
Acute (<1 mo)										
Isho, 2016	10 mWT	**CGS:** median 0.79 (range 0.23–1.04)	15 m, 2.5m, 2.5m	0 practice, 1 test trial, NR, stopwatch	Comfortable (barefoot), yes, no	Within-session	15	0.95 (0.86–0.98)	-	Adequate
Acute (<1 mo) and subacute (1–6 mo)										
English, 2007	5 mWT	**CGS**: individual therapy 0.37±0.40; circuit class therapy 0.41±0.43	10 m, 3m, 2m	0 practice,^1^ 1 test trial, behind, stopwatch	Comfortable, yes, no	N/A, video-taped trials re-scored	10	Intra-R: 1.00 (NR)	-	Adequate
Fulk, 2008	5 mWT	**CGS:** 0.45±0.30	9 m, 2 m, 2 m	0 practice, 1 test trial,	Comfortable, yes, yes^4^	1–3d	35, All Subjects	0.86 (0.68–0.94)	MDC_90_ = 0.30m/s MDC_90_ = 0.26m/s ^2^	Very Good
	**CGS:** 0.26±0.18		NR, stopwatch			13, Require physical assistance	0.97 (0.91–0.99)	MDC_90_ = 0.07m/s MDC_90_ = 0.12m/s ^2^
	**CGS:** 0.56±0.30					22, no physical assistance	0.80 (0.41–0.93)	MDC_90_ = 0.36m/s MDC_90_ = 0.31m/s ^2^
	**CGS:** 0.36±0.25					28, Require an assistive device	0.91 (0.71–0.97)	MDC_90_ = 0.18m/s MDC_90_ = 0.17m/s ^2^
Subacute (1–6 mo)										
Lam, 2010	6 mWT	**CGS:** 0.46±0.29	10 m, 2 m, 2 m	1 practice (day before),	Comfortable, yes, no	Within session (10 min)	45	Intra-R: 0.99 (*p* = 0.00)	-	Adequate
		(0.06–1.14)^2^ *first trial^1^*		1 test trial, NR, stopwatch		1d (same time)	45	Inter-R: 0.99 (*p* = 0.00)	MDC_90_ = 0.07m/s ^2^
Stephens, 1999	6 mWT	**CGS:** 0.75±0.24^2^	10 m, 2 m, 2 m **(carpet)**	1 practice, 1 test trial,	Comfortable, yes, no	1d, same time Note: 5 min	24	0.94 (NR) r = 0.95 (NR)	MDC_90_ = 0.14m/s^2^	Adequate
		**CGS:** 0.79±0.23^2^	**(parquetry)**	beside, stopwatch	Comfortable, yes, no	rest between 4 trials	24	0.97 (NR) r = 0.97 (NR)	MDC_90_ = 0.09m/s^2^
	**FGS:** 1.08±0.37^2^	**(carpet)**		Maximum, yes, no	completed in random	24	0.95 (NR) r = 0.95 (NR)	MDC_90_ = 0.19m/s^2^
	**FGS:** 1.11±0.42^2^	**(parquetry)**		Maximum, yes, no	order	24	0.94 (NR) r = 0.94 (NR)	MDC_90_ = 0.24m/s^2^
Høyer, 2014	10 mWT	**FGS:** 0.25±0.15	trial 1 : 13 m, 3 m, 0 m trial 2 : 10, 0 m, 0 m	0 practice, 1 test trial, behind, stopwatch	Maximum, no^7^, yes^5^	Within session	21, Baseline	0.96 (NR)	MDC_90_ = 0.07m/s^2^ MDC_95_ = 0.09 m/s	Adequate
Subacute (1–6 mo) and chronic (>6 mo)										
Høyer, 2014	10 mWT	**FGS:** 0.25±0.15	trial 1 : 13 m, 3 m, 0 m trial 2 : 10, 0 m, 0 m	0 practice, 1 test trial, behind, stopwatch	Maximum, no^7^, yes^5^	Within session	21, after 11 weeks of gait training	0.99 (NR)	MDC_90_ = 0.07m/s^2^ MDC_95_ = 0.08 m/s	Adequate
Chronic (>6 mo)										
Flansjber, 2005	10 mWT	**CGS:** 0.89±0.3 (0.4–1.4) **FGS:** 1.3±0.5 (0.5–2.2)	14 m, 2 m, 2 m,	0 practice, 3 test trials, NR, stopwatch	Comfortable, yes, no	7d (same time) Mean of 3 trials	50	0.94 (0.90–0.97)	MDC_90_ = 0.17m/s^2^ SEM (SEM%) = 0.07 m/s (7.9 m/s) SRD% (95% CI) = 22 m/s (–0.15 m/s–0.25 m/s)	Very Good
					Maximum, yes, no	7d (same time) Mean of 3 trials	50	0.97 (0.95–0.98)	MDC_90_ = 0.20m/s^2^ SEM (SEM%) = 0.08m/s (5.7 m/s) SRD% (95% CI) = 16 m/s (–0.21 m/s–0.22 m/s)
Faria, 2012	10mWT	Not Baseline: **CGS**	14 m, 2 m, 2 m,	1 practice, 3 test trials, NR, stopwatch	Comfortable, yes, no	1st trial: 1 min	16	Intra-R: 0.94 (*p*≤0.001) Inter-R: 0.96 (*p*≤0.001)	SEM (SEM%) = 0.05 m/s (5.01 m/s)	Very Good
		**Best of 3 trials:** 1.1±0.26				Mean of 2 trials: 1 min	16	Intra-R: 0.87 (*p*≤0.001) Inter-R: 0.97 (*p*≤0.001)	SEM (SEM%) = 0.07 m/s (6.40 m/s)
		**FGS, Best of 3 trials:** 1.4±0.36				Mean of 3 trials: 1 min	16	Intra-R: 0.95 (*p*≤0.001) Inter-R: 0.97 (*p*≤0.001)	SEM (SEM%) = 0.07 m/s (6.32 m/s)
						Best of 3 trials: 1 min	16	Intra-R: 0.92 (*p*≤0.001) Inter-R: 0.93 (*p*≤0.001)	SEM (SEM%) = 0.06 m/s (5.63 m/s)
						Worst of 3 trials:1 min	16	Intra-R: 0.88 (*p*≤0.001) Inter-R: 0.93 (*p*≤0.001)	SEM (SEM%) = 0.07 m/s (6.52 m/s)
					Maximum, yes, no	1^st^ trial: 1 min	16	Intra-R: 0.86 (*p*≤0.001) Inter-R: 0.91 (*p*≤0.001)	SEM (SEM%) = 0.14 m/s (9.86 m/s)
						Mean of 2 trials: 1 min	16	Intra-R: 0.92 (*p*≤0.001) Inter-R: 0.96 (*p*≤0.001)	SEM (SEM%) = 0.14 m/s (9.96 m/s)	(*Continued*)
						Mean of 3 trials: 1 min	16	Intra-R: 0.92 (*p*≤0.001) Inter-R: 0.97 (*p*≤0.001)	SEM (SEM%) = 0.14 m/s (9.62 m/s)
						Best of 3 trials: 1 min	16	Intra-R: 0.84 (*p*≤0.001) Inter-R: 0.92 (*p*≤0.001)	SEM (SEM%) = 0.14 m/s (9.87 m/s)
						Worst of 3 trials: 1 min	16	Intra-R: 0.83 (*p*≤0.001) Inter-R: 0.91 (*p*≤0.001)	SEM (SEM%) = 0.15 m/s (10.68 m/s)
Hiengkaew, 2012	10mWT	**CGS:** 0.51±0.30 (0.11–1.32)	14 m, 2 m, 2 m	NR, NR, NR, stopwatch	Comfortable, yes, no	5 to 10d, same time	61, All subjects	0.96 (0.92–0.98)	MDC_90_ = 0.14 m/s^2^ SEM = 0.06 m/s	Very Good
		**CGS:** 0.77±0.33 (0.33–0.77)				Note: 3–5 min rest between comfortable &	12^3^ PF tone: no ↑	0.96 (0.86–0.99)	MDC_90_ = 0.15 m/s^2^ SEM = 0.07 m/s
		**CGS:** 0.47±0.29 (0.11–1.18)				maximum trials	32^3^ PF tone: slight ↑	0.95 (0.89–0.98)	MDC_90_ = 0.15 m/s^2^ SEM = 0.06 m/s
	**CGS:** 0.39±0.19 (0.17–0.85)					17, MAS≥2^3^ PF tone: marked ↑	0.95 (0.81–0.99)	MDC_90_ = 0.10 m/s^2^ SEM = 0.03 m/s
		**FGS:** 0.62±0.40 (0.09–1.40)			Maximum, yes, no		61, All Subjects	0.98 (0.97–0.99)	MDC_90_ = 0.13 m/s^2^ SEM = 0.06 m/s
		**FGS:** 0.97±0.46 (0.46–0.97)					12^3^ PF tone: no ↑	0.99 (0.97–0.99)	MDC_90_ = 0.11 m/s^2^ SEM = 0.05 m/s
	**FGS:** 0.56±0.37 (0.10–1.48)					32^3^ PF tone: slight ↑	0.98 (0.95–0.99)	MDC_90_ = 0.12 m/s^2^ SEM = 0.05 m/s
		**FGS:** 0.47±0.27 (0.18–1.03)					17^3^ PF tone: marked ↑	0.99 (0.95–0.99)	MDC_90_ = 0.06 m/s^2^ SEM = 0.03 m/s
Peters, 2014	3 mWT	**CGS:** 0.27±0.11	3 m, 2 m, 2 m	1 practice, 1 test trial, NR, stopwatch	Comfortable, yes, no	Within-session rest provided as needed	12, household ambulators	0.97 (0.94–0.99)	MDC_90_ = 0.04^2^ MDC_95_ = 0.05 SEM = 0.04	Adequate
		**CGS:** 0.52±0.10					24, limited community ambulators	0.91 (0.85–0.94)	MDC_90_ = 0.07^2^ MDC_95_ = 0.08 SEM = 0.06
		**CGS:** 0.89±0.15					26, community ambulators	0.85 (0.77–0.90)	MDC_90_ = 0.14^2^ MDC_95_ = 0.16 SEM = 0.12
Acute (<1 mo), subacute (1–6 mo) and chronic (>6 mo)										
Cheng, 2020	10 mWT	**CGS:** 0.94±0.36	14 m, 2 m, 2 m	0 practice, 1 test trial, beside and slightly behind, stopwatch	Comfortable, yes, yes^6^	1–3d (same time)	20	0.83 (0.63–0.93)	MDC_90_ = 0.34 MDC_95_ = 0.40	Very Good

**Abbreviations:** 10 mWT, 10-metre walk test; 95% confidence interval; 3 mWT, 3-metre walk test; 5 mWT, 5-metre walk test; 6 mWT, 6-metre walk test; AD, acceleration distance; CGS, comfortable gait speed; COSMIN, COnsensus-based Standards for the selection of health Measurements Instruments; d, day(s); DD, Deceleration distance; FGS, fast gait speed; ICC, intraclass correlation coefficient; Inter-R, Inter-rater reliability; Intra-R, Intra-rater reliability; m, metres; m/s, metres per second; MDC, Minimal detectable change; min, minute(s); PF, ankle plantarflexors; mo, months; N/A, not applicable; NR, not reported; SEM, Standard error of measurement; SRD, Smallest real difference; TD, total distance. ^1^Data obtained from author. ^2^Computed from study data. ^3^Modified Ashworth Scale (MAS) was used to classify ankle plantar-flexor tone as: no increase (MAS = 0), slight increase (MAS = 1-1+), and marked increase (MAS≥2). ^4^Patient’s physical therapist determined the amount of physical assistance given. ^5^People dependent on 1 person to walk participated. A physical therapist secured the patient from behind by close manual support, holding the waistband, or by close presence and supervision, and only moved when the patient was in double support phase. ^6^Evaluator provided physical assistance at the waist to steady the person, if needed, but not to advance the foot. ^7^Orthoses were permitted if a prerequisite for safety.

### 3.7 Construct validity

Table 3 presents construct validity findings from 33 articles, including 118 correlation coefficients for relationships between measures of targeted constructs and performance on the 5 mCWT (27 correlations), 5 mFWT (1 correlation), 6 mCWT (2 correlations), 7 mCWT (4 correlations), 7 mFWT (4 correlations), 8 mCWT (5 correlations), 10 mCWT (61 correlations), and 10 mFWT (11 correlations), and 12 mCWT (3 correlations). Across phases of stroke recovery, the majority of correlation coefficients were evaluated for the chronic phase post-stroke (56 correlations; 46%). Of the 118 correlations, 3 were predictive in nature, demonstrating the ability of the 10 mCWT to predict physical activity (*r* = 0.60–0.66) (Alzahrani et al., 2009; Fulk et al., 2010).

**Table 3:** Construct Validity (*n* = 33 articles; 41 protocols evaluated)

Walk Test	ICF Classification	Construct	Measure	Results Pearson r (*P*-value, n) Spearman *ρ* (*P*-value, n)
Acute (< 1mo)				
10 mCWT	Body Function	Balance	Trunk impairment scale (coordination)	*ρ*= 0.62 (*P* < 0.05, *n* = 15) (Isho &Usuda, 2016)
			Trunk impairment scale (total)	*ρ*= 0.43 (NS, *n* = 15) (Isho &Usuda, 2016)
			Trunk impairment scale (dynamic sitting balance)	*ρ*= 0.10 (NS, *n* = 15) (Isho &Usuda, 2016)
		Strength: hand-grip^1^	Dynamometer	r = 0.49 (*P* = 0.0002, *n* = 59) (Di Cesare et al., 2016)
		Strength: hand-grip^2^	Dynamometer	r = 0.24 (NS, *n* = 64) (Di Cesare et al., 2016)
		Strength: knee extensor^1^	Dynamometer	*ρ*= 0.26 (NS, *n* = 15) (Isho &Usuda, 2016)
		Strength: knee extensor^2^	Dynamometer	*ρ*= 0.16 (NS, *n* = 15) (Isho &Usuda, 2016)
	Activity	Balance	Short-form Berg balance scale	*ρ*= 0.34 (NS, *n* = 15) (Isho &Usuda, 2016)
		Mobility	Modified Rankin scale (30 days)	r = –0.51 (*P* < 0.0001, *n* = 77) (Di Cesare et al., 2016)
			Modified Rankin scale (90 days)	r = –0.50 (*P* < 0.0001, *n* = 75) (Di Cesare et al., 2016)
			Modified Rankin scale (7 days)	r = –0.31 (*P* = 0.0134, *n* = 64) (Di Cesare et al., 2016)
Acute (<1 mo) and subacute (1–6 mo)				
5 mCWT	Body Function	Motor function and basic	STREAM (initial assessment)	r = 0.74 (*P* < 0.0001, *n* = 63) (Ahmed et al., 2003)
	&Activity	mobility	STREAM (3 months)	r = 0.73 (*P* < 0.0001, *n* = 63) (Ahmed et al., 2003)
			STREAM (5 weeks)	r = 0.62 (*P* < 0.0001, *n* = 63) (Ahmed et al., 2003)
	Body Function	Motor function:	STREAM (3 months)	r = 0.64 (*P* < 0.001, *n* = 63) (Ahmed et al., 2003)
		upper extremity	STREAM (initial assessment)	r = 0.56 (*P* < 0.0001, *n* = 63) (Ahmed et al., 2003)
			STREAM (5 weeks)	r = 0.53 (*P* < 0.0001, *n* = 63) (Ahmed et al., 2003)
	Body Function	Motor function: lower extremity	STREAM (initial assessment)	r = 0.74 (*P* < 0.0001, *n* = 63) (Ahmed et al., 2003)
			STREAM (3 months)	r = 0.65 (*P* < 0.0001, *n* = 63) (Ahmed et al., 2003)
			STREAM (5 weeks)	r = 0.55 (*P* < 0.0001, *n* = 63) (Ahmed et al., 2003)
	Activity	Basic mobility	STREAM (initial assessment)	r = 0.83 (*P* < 0.0001, *n* = 63) (Ahmed et al., 2003)
			STREAM (3 months)	r = 0.76 (*P* < 0.0001, *n* = 63) (Ahmed et al., 2003)
			STREAM (5 weeks)	r = 0.65 (*P* < 0.0001, *n* = 63) (Ahmed et al., 2003)
6 mCWT	Body function	Strength: knee extensor	Dynamometer	*ρ*= 0.55 (*P* < 0.01, *n* = 45) (Lam et al., 2010)
	Activity	Comfortable walk speed	10 mWT	*ρ*= 0.99 (*P* < 0.01, *n* = 45) (Lam et al., 2010)
10 mCWT	Activity	Walk speed	6 MWT speed	r = 0.385 (*P* = 0.022, *n* = 64) (Morone et al., 2014)
10 mFWT	Body Function	Motor function: lower extremity	Fugl-Meyer assessment scale for the lower extremity (FMA-LE)	r = 0.62 (*P* < 0.001, *n* = 75) (Mitsutake et al., 2017)
Subacute (1–6 mo)				
5 mCWT	Activity	Physical activity	ActivPAL3 Micro- # steps at medium rate (80–99 steps/min)	*ρ*= 0.79 (*P* < 0.01, *n* = 30) (Ezeugwu &Manns, 2017)
			ActivPAL3 Micro- # steps at brisk rate (100–119 steps/min)	*ρ*= 0.74 (*P* < 0.01, *n* = 30) (Ezeugwu &Manns, 2017)
			ActivPAL3 Micro- mean steps/d	*ρ*= 0.61 (*P* < 0.01, *n* = 30) (Ezeugwu &Manns, 2017)
			ActivPAL3 Micro- stepping time (min)	*ρ*= 0.49 (*P* < 0.01, *n* = 30) (Ezeugwu &Manns, 2017)
			ActivPAL3 Micro- # steps at fastest rate (> 120 steps/min)	*ρ*= 0.47 (*P* < 0.01, *n* = 30) (Ezeugwu &Manns, 2017)
			ActivPAL3 Micro- # sit-to-stand transitions	*ρ*= 0.34 (NS, *n* = 30) (Ezeugwu &Manns, 2017)
			ActivPAL3 Micro- standing time (min)	*ρ*= 0.32 (NS, *n* = 30) (Ezeugwu &Manns, 2017)
			ActivPAL3 Micro- sedentary time (min)	*ρ*= –0.28 (NS, *n* = 30) (Ezeugwu &Manns, 2017)
			ActivPAL3 Micro- # steps at slow rate (60–79 steps/min)	*ρ*= 0.25 (NS, *n* = 30) (Ezeugwu &Manns, 2017)
			ActivPAL3 Micro- # steps at purposeful rate (40–59 steps/min)	*ρ*= 0.14 (NS, *n* = 30) (Ezeugwu &Manns, 2017)
			ActivPAL3 Micro- # steps at sporadic rate (20–39 steps/min)	*ρ*= –0.02 (NS, *n* = 30) (Ezeugwu &Manns, 2017)
		Walk distance	6 MWT	r = 0.89 (*P* < 0.000, *n* = 37) (Fulk et al., 2008)
Subacute (1–6 mo) and chronic (≥6 mo)				
5 mCWT	Body function	Balance self-efficacy	Activities-specific balance confidence scale	*ρ*= 0.46 (95% CI 0.28–0.61, *n* = 89) (Salbach et al., 2006)
5 mFWT	Body function	Balance self-efficacy	Activities-specific balance confidence scale	*ρ*= 0.49 (95% CI 0.31–0.63, *n* = 89) (Salbach et al., 2006)
10 mCWT	Body function	Strength: knee extensor	Dynamometer	r = 0.18 (NS, *n* = 48, absolute values) (Severinsen et al., 2011)
				r = 0.31 (*P* < 0.05, *n* = 48, normalized values) (Severinsen et al., 2011)
Chronic (≥6 mo)				
5 mCWT	Activity	Physical activity	ActivPAL–mean standing time (mins/d) over 5 days	*ρ*= 0.50 (*P* = 0.043 *n* = 17) (Salbach et al., 2013)
	Participation	Participation	Stroke impact scale - participation (%)	*ρ*= 0.48 (*P* = 0.049, *n* = 17) (Salbach et al., 2013)
10 mCWT	Body function	Aerobic capacity	VO_2peak_ (cycle ergometer)	r = 0.33 (*P* < 0.05, *n* = 48, absolute values) (Severinsen et al., 2011)
				r = 0.53 (*P* < 0.05, *n* = 48, normalized values) (Severinsen et al., 2011)
			VO_2peak_ (treadmill)	*ρ*= NR (NS, *n* = 8) (Ovando et al., 2011)
		Strength	Stroke impact scale-strength	r = 0.64 (*P* < 0.001, *n* = 92) (Khanittanuphong &Tipchatyotin, 2017)
		Strength: lower limb	Motricity Index	NR = 0.62 (*P* < 0.01, *n* = 46) (Lee et al., 2015)
		Strength: knee flexor	Dynamometer	r = 0.80 (*P* < 0.05, *n* = 12) (Nasciutti-Prudente et al., 2009)
			Dynamometer	r = 0.61 (*P* < 0.01, *n* = 50) (Flansbjer et al., 2006)
		Strength: knee extensor	Dynamometer	r = 0.61 (*P* < 0.01, *n* = 50) (Flansbjer et al., 2006)
			Dynamometer	r = 0.34 (NS, *n* = 12) (Nasciutti-Prudente et al., 2009)
		Strength: quadriceps	Dynamometer	r = 0.35 (*P* < 0.01, *n* = 63, normalized) (Liu-Ambrose et al., 2007)
		Strength: hip flexor	Dynamometer	r = 0.75 (*P* < 0.05, *n* = 12) (Nasciutti-Prudente et al., 2009)
		Strength: hip extensor	Dynamometer	r = 0.53 (NS, *n* = 12) (Nasciutti-Prudente et al., 2009)
		Strength: ankle dorsiflexor	Dynamometer	r = 0.50 (NS, *n* = 12) (Nasciutti-Prudente et al., 2009)
		Strength: ankle plantar-flexor	Dynamometer	r = 0.58 (*P* < 0.05, *n* = 12) (Nasciutti-Prudente et al., 2009)
	Activity	Balance	360-degree turn (turn time)	r = –0.76 (*P* < 0.01, *n* = 38) (Kobayashi et al., 2015)
			Berg balance scale (items 1–12 + item 13 (nonparetic leg in front)+item 14 (SLS on paretic leg))	p = 0.72 (*P* = 0.001, *n* = 63) (Kwong et al., 2016)
			Berg balance scale (items 1–12 + item 13 (paretic leg in front)+item 14 (SLS on paretic leg)	p = 0.70 (*P* = 0.001, *n* = 63) (Kwong et al., 2016)
			Berg balance scale	NR = 0.69 (*P* < 0.01, *n* = 46) (Lee et al., 2015)
			Stroke impact scale- mobility	r = 0.64 (*P* < 0.001, *n* = 92) (Khanittanuphong &Tipchatyotin, 2017)
			360-degree turn (steps in turn)	r = –0.59 (*P* < 0.01, *n* = 38) (Kobayashi et al., 2015)
		Hand function	Stroke impact scale- hand function	r = 0.52 (*P* < 0.001, *n* = 92) (Khanittanuphong &Tipchatyotin, 2017)
		Capacity for activities of	FIM	r = 0.63 (*P* < 0.01, *n* = 50) (Frost et al., 2015)
		daily living	IADL Questionnaire	r = 0.50 (*P* < 0.01, *n* = 50) (Frost et al., 2015)
		Physical activity	Activity counts (sum of number of steps walked, stairs, number of transitions)	r = 0.66 (*P* < 0.001, *n* = 42) *predictive* (Alzahrani et al., 2009)
			SAM- mean steps/day	r = 0.65 (*P* = 0.003, *n* = 19) *predictive (Fulk et al., 2010)*
			SAM- Peak Activity Index (steps/min)	r = 0.64 (*P* < 0.01, *n* = 49) (Mudge &Stott, 2009)
			Time on feet (sum of minutes walking, stairs, standing, sit to stand)	r = 0.60 (*P* < 0.001, *n* = 42) *predictive* (Alzahrani et al., 2009)
			SAM- means steps/day	*ρ*= 0.55 (*P* < 0.01, *n* = 49) (Mudge &Stott, 2009)
			SAM- # steps at high rate^4^	*ρ*= 0.54 (*P* < 0.01, *n* = 49) (Mudge &Stott, 2009)
			SAM- # steps at low rate^3^	r = 0.46 (*P* < 0.01, *n* = 49) (Mudge &Stott, 2009)
			Physical activity scale for individuals with physical disabilities	r = 0.42 (*P* < 0.01, *n* = 50) (Frost et al., 2015)
			Current PA level (PASIPD Score) in MET-h/day	r = 0.26 (*P* < 0.05, *n* = 63) (Liu-Ambrose et al., 2007)
		Walk distance	6 MWT	NR = 0.89 (*P* < 0.01, *n* = 46) (Lee et al., 2015)
		Community walking capacity	Total time taken to walk 300m community route	*ρ*= –0.88 (*P* < 0.0001, *n* = 28) (Taylor et al., 2006)
	Participation	Participation	Stroke impact scale- Participation (%)	r = 0.57 (*P* < 0.01, *n* = 50) (Flansbjer et al., 2006)
			Stroke impact scale- participation	r = 0.56 (*P* < 0.001, *n* = 92) (Khanittanuphong &Tipchatyotin, 2017)
10 mCWT-	Activity	Mobility	Timed Up and Go (session 2)	r = –0.84 (*P* < 0.001, *n* = 50) (Flansbjer et al., 2005)
session 1		Stair function	Stair climbing- ascend (session 2)	r = –0.81 (*P* < 0.001, *n* = 50) (Flansbjer et al., 2005)
			Stair climbing- descend (session 2)	r = –0.82 (*P* < 0.001, *n* = 50) (Flansbjer et al., 2005)
		Fast walk speed	10 mFWT (session 2)	r = 0.92 (*P* < 0.001, *n* = 50) (Flansbjer et al., 2005)
		Walk distance	6 MWT (session 2)	r = 0.89 (*P* < 0.001, *n* = 50) (Flansbjer et al., 2005)
10 mFWT	Body function	Strength: knee flexor	Dynamometer	r = 0.65 (*P* < 0.01, *n* = 50) (Flansbjer et al., 2006)
		Strength: knee extensor	Dynamometer	r = 0.67 (*P* < 0.01, *n* = 50) (Flansbjer et al., 2006)
	Body function	Aerobic capacity	VO_2peak_ (treadmill)	*ρ*= NR (NS, *n* = 8) (Ovando et al., 2011)
	Activity	Mobility	Timed Up and Go	r = 0.91 (*P* < 0.01, *n* = 27) (Huo et al., 2009)
	Participation	Participation	Stroke impact scale- Participation (%)	r = 0.57 (*P* < 0.01, *n* = 50) (Flansbjer et al., 2006)
10 mFWT-	Activity	Mobility	Timed Up and Go (session 2)	r = –0.91 (*P* < 0.001, *n* = 50) (Flansbjer et al., 2005)
session 1		Stair function	Stair climbing- ascend (session 2)	r = –0.84 (*P* < 0.001, *n* = 50) (Flansbjer et al., 2005)
			Stair climbing- descend (session 2)	r = –0.87 (*P* < 0.001, *n* = 50) (Flansbjer et al., 2005)
		Comfortable walk speed	10 mCWT (session 2)	r = 0.88 (*P* < 0.001, *n* = 50) (Flansbjer et al., 2005)
		Walk distance	6 MWT (session 2)	r = 0.95 (*P* < 0.001, *n* = 50) (Flansbjer et al., 2005)
12 mCWT	Body function	Aerobic capacity	VO_2max_	r = 0.47 (*P* < 0.05, *n* = 35) (Wang et al., 2014)
		Strength: knee extensor (90-degree torque)	Dynamometer	r = 0.62 (*P* < 0.05, *n* = 35) (Wang et al., 2014)
		Strength: knee extensor (60-degree torque)	Dynamometer	r = 0.62 (*P* < 0.05, *n* = 35) (Wang et al., 2014)
Acute (< 1 mo), subacute (1–6 mo), and Chronic (≥6 mo)				
7 mCWT	Body function	Strength: knee extensor	Lido Active Rehabilitation System	r = 0.67 (*P* < 0.01, *n* = 14) (Bohannon &Walsh, 1992)
		(peak torque)	Lido Active Rehabilitation System	r = 0.62 (*P* < 0.01, *n* = 18) (Bohannon &Puharic, 1992)
		Strength: knee extensor (torque)	Lido Active Rehabilitation System	r = 0.75 (*P* = 0.000, *n* = 20) (Bohannon, 1992)
		Strength: knee extensor (peak power)	Lido Active Rehabilitation System	r = 0.75 (*P* = 0.000, *n* = 20) (Bohannon, 1992)
7 mFWT	Body function	Strength: knee extensor	Lido Active Rehabilitation System	r = 0.76 (*P* < 0.01, *n* = 14) (Bohannon &Walsh, 1992)
		(peak torque)	Lido Active Rehabilitation System	r = 0.64 (*P* < 0.01, *n* = 18) (Bohannon &Puharic, 1992)
		Strength: knee extensor (torque)	Lido Active Rehabilitation System	r = 0.74 (*P* = 0.000, *n* = 20) (Bohannon, 1992)
		Strength: knee extensor (peak power)	Lido Active Rehabilitation System	r = 0.74 (*P* = 0.000, *n* = 20) (Bohannon, 1992)
8 mCWT	Body function	Strength: knee extensor (force)	Dynamometer	r = 0.60 (*P* < 0.01, *n* = 26); (Bohannon, 1991)
				r = 0.62 (*P* < 0.001, *n* = 26, normalized with body weight) (Bohannon, 1991)
		Strength: knee extensor (measured torque)	Isokinetic dynamometer	r = 0.65 (*P* < 0.001, *n* = 26); (Bohannon, 1991)
				r = 0.68 (*P* < 0.001, *n* = 26, normalized with body weight) (Bohannon, 1991)
		Strength: knee extensor (percent of body weight)	Dynamometer	r = 0.67 (*P* < 0.01, *n* = 20) (Bohannon, 1991)
10 mCWT	Body function	Strength (baseline and re-test)	Stroke impact scale-strength	r = 0.27 (*P* = 0.232, *n* = 21) (Cheng et al., 2020)
				r = 0.29 (*P* = 0.232, *n* = 20) (Cheng et al., 2020)
	Activity	Walk distance (baseline and re-test)	6 MWT (15 m walkway)	r = 0.80 (*P* = 0.000, *n* = 21) (Cheng et al., 2020)
				r = 0.94 (*P* = 0.000, *n* = 20) (Cheng et al., 2020)
			6 MWT (30 m walkway)	r = 0.80 (*P* = 0.000, *n* = 21) (Cheng et al., 2020)
				r = 0.91 (*P* = 0.000, *n* = 20) (Cheng et al., 2020)

**Abbreviations:** 10 mCWT, 10-metre comfortable walk test; 10 mFWT, 10-metre fast walk test; 10 mWT, 10-metre walk test; 12 mCWT, 12-metre comfortable walk test; 5 mCWT, 5-metre comfortable walk test; 5 mFWT, 5-metre fast walk test; 6 mCWT, 6-metre comfortable walk test; 6 MWT, 6-minute walk test; 7 mCWT, 7-metre comfortable walk test; 7 mFWT, 7-metre fast walk test; 8 mCWT, 8-metre comfortable walk test; d, day; FIM, functional independence measure; IADL, instrumental activities of daily living; m, metres; MET-h, metabolic equivalent hours; min, minute(s); mo, month(s); NR, not reported; NS, not significant; PA, physical activity; PASIPD, Physical Activity Scale for Individuals with Physical Disabilities; SAM, StepWatch Activity Monitor; STREAM, Stroke Rehabilitation Assessment of Movement. ^1^Paretic side. ^2^Non-paretic side. ^3^Number of steps at a low rate is defined as < 30 steps per minute.^4^Number of steps at a high rate is defined as > 60 steps per minute.

### 3.8 Sensitivity to change

Table 4 presents estimates of sensitivity to change reported in 3 articles. Large ES/SRM were observed for the 5 mCWT, and medium ES and large SRM for the 5 mFWT, 10 mCWT, and 10 mFWT in people with acute and subacute stroke (Ahmed et al., 2003; English et al., 2006; Salbach et al., 2001).

**Table 4:** Sensitivity to Change (N = 3 articles)

First Author, Year	Walk Test, Measurement Tool, Location	Distance (m) TD: AD: DD:	Pace	Time Interval between walk tests	N	Effect Size (ES) Standardized Response Mean (SRM)
Acute (< 1 mo) &Sub-Acute (1–6 mo)						
Salbach, 2001	5 mWT	**TD:** 9 m	Comfortable	Time between 1 week and	50	ES = 0.83
	Stopwatch	**AD:** 2 m		5 weeks post- acute stroke		SRM (95% CI) = 1.22 (0.93–1.50)
	Indoor	**DD:** 2 m	Fast			ES = 0.66
						SRM (95% CI) = 1.00 (0.68–1.30)
	10 mWT	**TD:** 14m	Comfortable			ES = 0.74
	Stopwatch	**AD:** 2 m				SRM (95% CI) = 0.92 (0.64–1.18)
	Indoor	**DD:** 2 m	Fast			ES = 0.55
						SRM (95% CI) = 0.83 (0.52–1.12)
Ahmed, 2003	5 mWT	**TD:** 9 m	Comfortable	Time between 1 week and	63	SRM (95% CI) = 1.05 (0.79–1.24)
	Stopwatch	**AD:** 2 m		5 weeks post-acute stroke
	Indoor	**DD:** 2 m
				Time between 5 weeks and		SRM (95% CI) = –0.17 (–0.13–0.43)
				3 months post-acute stroke
				Time between 1 week and		SRM (95% CI) = 1.15 (0.80–1.43)
				3 months post-acute stroke
English, 2006	5 mWT	**TD:** 10 m	Comfortable	Time between admission	61	ES = 0.81
	Stopwatch	**AD:** 3 m		and discharge from in-patient
	Indoors	**DD:** 2 m		rehabilitation (56±38 days)

**Abbreviations:** 10 mWT, 10-metre walk test; 95% CI, 95% confidence interval; 5 mWT, 5-metre walk test; m, metres; AD, acceleration distance; DD, deceleration distance; ES, effect size; mo, month(s); SRM, standardized response mean; TD, total distance.

Table 5 summarizes reliability, measurement error, sensitivity to change, and construct validity findings by walk test and recovery phase post-stroke.

**Table 5:** Reliability, Measurement Error, Sensitivity to Change, and Construct Validity Findings by Walk Test and Recovery Phase Post-stroke^1^

Walk Test	Reliability Coefficient			MDC_90_, m/s and ES or SRM			Constructs Correlated with Walk Test Performance		
	(# Articles)			(# Articles)			(# Correlations with *P* < 0.05)		
	Acute	Subacute	Chronic	Acute	Subacute	Chronic	Acute	Subacute	Chronic
3 mCWT			0.85–0.97 (1)			0.04–0.14 (1)
5 mCWT	1.00 (1)^2^	1.00 (1)^2^		MDC_90_: 0.07–0.36 (1)	MDC_90_: 0.07–0.36 (1)		Motor function and basic mobility (3)	Motor function and basic mobility (3)	Physical activity (1)
	0.80–0.97 (1)	0.80–0.97 (1)		ES: 0.81–0.83 (2)	ES: 0.81–0.83 (2)		Motor function-U (3)	Motor function-U (3)	Participation (1)
				SRM: 1.05^3^–1.22 (2)	SRM: 1.05^3^–1.22 (2)		Motor function-L (3)	Motor function-L (3)
							Basic mobility (3)	Basic mobility (3)
								Physical activity (5)
								Walk distance (1)
								Balance self-efficacy (1)
5 mFWT				ES: 0.66 (1)	ES: 0.66 (1)			Balance self-efficacy (1)	Balance self- efficacy (1)
				SRM: 1.00 (1)	SRM: 1.00 (1)
6 mCWT		0.99 (1)^4^			MDC_90_: 0.07–0.14 (2)		Strength-L (1)	Strength-L (1)
		0.94–0.99 (2)					Comfortable walk speed (1)	Comfortable walk speed (1)
6 mFWT		0.94–0.95 (1)			MDC_90_: 0.19–0.24 (1)
7 mCWT							Strength-L (4)	Strength-L (4)	Strength-L (4)
7 mFWT							Strength-L (4)	Strength-L (4)	Strength-L (4)
8 mCWT							Strength-L (4)	Strength-L (4)	Strength-L (4)
10 mCWT	0.83–0.95 (2)	0.83 (1)	0.87–0.95 (1)^2^	MDC_90_: 0.34 (1)	MDC_90_: 0.34 (1)	MDC_90_: 0.10–0.34 (3)	Balance (1)	6-minute walk test walking speed (1)	Aerobic capacity (2)
			0.93–0.97 (1)^4^	ES: 0.74 (1)	ES: 0.74 (1)		Strength-U (1)	Strength-L (1)	Strength (1)
			0.83–0.96 (3)	SRM: 0.92 (1)	SRM: 0.92 (1)		Mobility (3)	Walk distance (2)	Strength-L (8)
							6-minute walk test walking speed (1)		Balance (6)
							Walk distance (2)		Hand function (1)
									Capacity for activities of daily living (2)
									Physical activity (9)
									Walk distance (4)
									Community walking capacity (1)
									Participation (2)
									Mobility (1)
									Stair function (2)
									Fast walk speed (1)
10 mFWT	0.96–0.99 (2)	0.97–0.99 (3)	ES: 0.55 (1)	MDC_90_: 0.07 (2)	MDC_90_: 0.06–0.20 (3)	Motor function-L (1)	Motor function-L (1)	Strength-L (2)
				SRM: 0.83 (1)	ES: 0.55 (1)				Mobility (2)
				SRM: 0.83 (1)					Participation (1)
									Stair function (2)
									Comfortable walk speed (1)
									Walk distance (1)
12 mCWT									Aerobic capacity (1)
Strength-L (2)

**Abbreviations:** 3 mCWT, 3-metre comfortable walk test; 5 mCWT, 5-metre comfortable walk test; 5 mFWT, 5-metre fast walk test; 6 mCWT, 6-metre comfortable walk test; 6 mFWT, 6-metre fast walk test; 7 mCWT, 7-metre comfortable walk test; 7 mFWT, 7-metre fast walk test; 8 mCWT, 8-metre comfortable walk test; 10 mCWT, 10-metre comfortable walk test; 10 mFWT, 10-metre fast walk test; 12 mCWT, 12-metre comfortable walk test; ES, effect size; L, lower extremity; MDC_90_, minimal detectable change at the 90% confidence level; SRM, standardized response mean; U, upper extremity. ^1^Results from studies that included participants in multiple phases of stroke recovery were listed under all phases. ^2^Intra-rater reliability. ^3^1–5 weeks post-stroke. ^4^Inter-rater reliability.

## 4 Discussion

This novel review provides a comprehensive synthesis of existing literature on measurement properties for distance-limited walk tests in people with stroke. The results are extensive which makes it challenging to understand how they might inform the selection of a distance-limited walk test to measure gait speed post-stroke in clinical practice. We therefore offer the following framework to guide decision-making that integrates systematic review findings.

The extensive evidence presented in this review can help guide the selection of a distance-limited walk test for clinical use post-stroke based on principles of measurement and generalizability, the influence of protocol elements on performance, and available resources (e.g., space). The first measurement principle guiding selection is an understanding that reliability is a prerequisite of validity (Streiner DL et al., 2014). One must first choose a walk test protocol that has demonstrated excellent reliability indicated by not only the ICC value, but also the lower limit of the 95% CI, and, secondarily, evidence of construct validity in the ‘population of interest’. Walking speed is a temporal-distance parameter of gait, not an abstract concept. Validity evidence increases our understanding of how strongly gait speed relates to impairments, activity limitations and participation restrictions (World Health Organization, 2001), and helps us to appreciate its relevance to human functioning, rehabilitation outcomes, and patient-centered goals.

The second principle guiding the selection of a distance-limited walk test for clinical use post-stroke relates to the generalizability of evidence to a particular clinical population (also known as external validity). If one’s clinical practice involves communication and/or program evaluation of walk test performance across acute care, and inpatient and outpatient rehabilitation settings (i.e., the care continuum), then ideally one will choose a distance-limited walk test with evidence of excellent reliability and validity in people with acute, subacute, and chronic stroke. If clinical use of walk test performance is limited to a single practice setting, one could select a test that is reliable and valid among patients seen in that setting alone. For clinical practice along the care continuum post-stroke, review findings reveal that the 10 mCWT is the only test with evidence of excellent reliability and construct validity in people with acute, subacute, and chronic stroke. Comfortable gait speed measured using the 10 mCWT consistently relates to balance and strength impairments, and mobility/walking limitations across settings; and participation in activities of daily living, physical activity, and other meaningful activities relevant to the out-patient setting (Lang et al., 2011) in people with chronic stroke. If one’s clinical practice is limited to treating people within 6 months post-stroke (acute and subacute phases), then the 5 mCWT is an excellent alternative, particularly for settings that cannot accommodate the 10 mCWT walkway length, given the evidence from this review of excellent reliability of the 5 mCWT and associations between 5 mCWT performance and important physical rehabilitation outcomes, such as motor function and basic mobility. Once reliability and validity evidence in the population of interest, and available space have been considered, a tertiary measurement consideration is sensitivity to change defined as the ability of a measure to detect change in the construct of interest (Cohen, 1977). Effect size/SRM estimates of sensitivity to change were large for the 5 mCWT and medium-to-large for the 10 mCWT in people with acute and subacute stroke (Ahmed et al., 2003; English et al., 2006; Salbach et al., 2001), reflecting the ability of both tests to capture change in walking capacity when individuals are likely participating in rehabilitation (Hall RE et al., 2018).

Generally, the walk test protocol, including instructions, acceleration/deceleration and timed distances, timing method, allowance for evaluator assistance and use of mobility devices, that is selected for clinical practice, should be identical to the one used in the reliability study supporting its use. Interestingly, review findings support an excellent level of reliability based on the ICC point estimate and lower 95% CI limit of diverse walk test protocols that did not allow physical assistance to walk (English et al., 2007; Faria et al., 2012; Flansbjer et al., 2005; Hiengkaew et al., 2012; Isho & Usuda, 2016; Lam et al., 2010; Peters et al., 2014; Stephens & Goldie, 1999). These protocols included walkways of 3 m (Peters et al., 2014), 5 m (English et al., 2007), 6 m (Lam et al., 2010; Stephens & Goldie, 1999), and 10 m (Faria et al., 2012; Flansbjer et al., 2005; Hiengkaew et al., 2012; Isho & Usuda, 2016) traversed at a comfortable pace, and 6 m (Stephens & Goldie, 1999) and 10 m (Faria et al., 2012; Flansbjer et al., 2005; Hiengkaew et al., 2012) walked at a fast pace; acceleration/deceleration distances of 2.0 m (Faria et al., 2012; Flansbjer et al., 2005; Hiengkaew et al., 2012; Lam et al., 2010; Peters et al., 2014; Stephens & Goldie, 1999) or 2.5 m (English et al., 2007; Isho & Usuda, 2016); 0 or 1 practice trial and 1 test trial (English et al., 2007; Isho & Usuda, 2016; Lam et al., 2010; Stephens & Goldie, 1999), as well as the mean of 2 or 3 trials (Faria et al., 2012; Flansbjer et al., 2005) or the maximum of 3 trials (Faria et al., 2012); and individuals with variable levels of plantar flexor tone (Hiengkaew et al., 2012) and community ambulation (Peters et al., 2014). It appears that, regardless of the protocol, any standardized distance-limited test to evaluate walking speed in people with stroke not requiring assistance is highly reliable. However, it is important that selected walk tests be compared to tests with the same testing distance and protocol, as results from only one study of people chronic stroke (Lam et al., 2010) showed that walkway distance did not affect walking speed. One cannot assume that these results apply to people with acute and subacute stroke, populations that are often seen in rehabilitation settings with less stable walking capacity compared to people with chronic stroke (Christensen et al., 2008; Schepers et al., 2006).

Excellent reliability based on ICC magnitude alone was also observed for a small number of walk test protocols (i.e., 5 mCWT, 10 mCWT, and 10 mFWT) in studies of very good or adequate quality that allowed the evaluator to provide physical assistance (Cheng et al., 2020; Fulk & Echternach, 2008; Høyer et al., 2014), with lower 95% CI limits in the acceptable range (Cheng et al., 2020; Fulk & Echternach, 2008). These findings are extremely relevant to acute and inpatient rehabilitation settings in which a substantial proportion of people post-stroke require assistance to walk (Hall RE et al., 2018). Healthcare professionals in these settings should consider adopting a protocol that allows the evaluator to provide physical assistance at the waist (Cheng et al., 2020; Høyer et al., 2014), but not to advance the lower extremity (Cheng et al., 2020). In fact, in people with acute and subacute stroke walking at slow speeds (e.g., mean ∼0.25 m/s), the reliability of walk test protocols evaluated is excellent and MDC_90_ values are small (0.07 or 0.12 m/s) (Fulk & Echternach, 2008; Høyer et al., 2014).

This review revealed gaps in the literature. Evidence for the reliability of the 5 mF-, 7 mC-, 7 mF-, 8 mC-, and 12 mCWT, for measurement error of the 5 mF-, 6 mF-, 7 mC-, 7 mF-, 8 mC-, and 12 mCWT, and for the construct validity of the 3 mC-, 6 mF-, and 8 mFWT, ideally across the care continuum, was lacking. Despite recommendations for the use of the 10 mCWT in clinical (Otterman et al., 2017; Sullivan et al., 2013; Teasell et al., 2020) and research (Kwakkel et al., 2017) settings, and its popularity in research studies (Salbach et al., 2014), there was limited research evaluating test-retest reliability and measurement error of this test in people with acute or subacute stroke. Furthermore, while some guidelines promote the 6-metre walk test for neurologic populations (Moore et al., 2018), our review found that evidence for reliability of this test was limited to the subacute stage, and the precision of the estimates is unknown because CIs were not reported (Lam et al., 2010; Stephens & Goldie, 1999). The vast majority of studies included in this review had limited applicability to rehabilitation settings as they enrolled people who walked faster than 0.4 m/s. Studies targeting people who walk slowly and may require assistance to walk, deficits commonly seen in acute care and inpatient rehabilitation settings (Hall RE et al., 2018), are needed.

This review has some limitations. Due to the extensive literature in this area and finite resources, we were unable to include evidence of validity for all constructs, studies of minimal clinically important change, or a more current review. More recent publications may address some of the gaps we identified. Although only one reviewer completed full text screening, data extraction and critical appraisal, extensive training and verification of data were undertaken. The review was comprehensive given the large number of databases searched and inclusion of any study reporting associations with gait speed for evidence of validity.

## 5 Conclusions

The 10 mCWT is the only measure demonstrating excellent reliability and construct validity across the care continuum post-stroke, and sensitivity to change in people with acute and subacute stroke. The 5 mCWT demonstrates excellent reliability, construct validity, and sensitivity to change in acute and subacute phases of stroke recovery. Despite wide variations, the majority of protocols for distance-limited tests have excellent reliability, and evidence of validity indicated by associations with important physical rehabilitation outcomes, even in people who require assistance to walk. Review findings provide guidance for future research and improved quality of reporting.
